# Respiratory Rhythm, Autonomic Modulation, and the Spectrum of Emotions: The Future of Emotion Recognition and Modulation

**DOI:** 10.3389/fpsyg.2020.01980

**Published:** 2020-08-14

**Authors:** Ravinder Jerath, Connor Beveridge

**Affiliations:** Charitable Medical Healthcare Foundation, Augusta, GA, United States

**Keywords:** respiration, respiratory rhythm, emotion, emotion recognition, autonomic nervous system, health monitoring

## Abstract

Pulmonary ventilation and respiration are considered to be primarily involved in oxygenation of blood for oxygen delivery to cells throughout the body for metabolic purposes. Other pulmonary physiological observations, such as respiratory sinus arrhythmia, Hering Brewer reflex, cardiorespiratory synchronization, and the heart rate variability (HRV) relationship with breathing rhythm, lack complete explanations of physiological/functional significance. The spectrum of waveforms of breathing activity correlate to anxiety, depression, anger, stress, and other positive and negative emotions. Respiratory pattern has been thought not only to be influenced by emotion but to itself influence emotion in a bi-directional relationship between the body and the mind. In order to show how filling in gaps in understanding could lead to certain future developments in mind–body medicine, biofeedback, and personal health monitoring, we review and discuss empirical work and tracings to express the vital role of bodily rhythms in influencing emotion, autonomic nervous system activity, and even general neural activity. Future developments in measurement and psychophysiological understanding of the pattern of breathing in combination with other parameters such as HRV, cardiorespiratory synchronization, and skin conductivity may allow for biometric monitoring systems to one day accurately predict affective state and even affective disorders such as anxiety. Better affective prediction based on recent research when incorporated into personal health monitoring devices could greatly improve public mental health by providing at-home biofeedback for greater understanding of one’s mental state and for mind–body affective treatments such as breathing exercises.

## Introduction

William James, known as the “Father of American Psychology,” once said, “For us, emotion dissociated from all bodily feeling is inconceivable” (James, 1884). In this statement, he has identified the intimate relationship between the body and the mind. Although there is no scientific consensus on an exact definition (Celeghin et al., 2017), the concept of emotion consists of a complex range of phenomenological and physiological states, which include neurobiological components, visceral reactions, bodily responses/behaviors, and of course, often powerful feelings (Purves et al., 2018), and can be described as an adaptive, patterned neural response to external circumstances that can be found at all levels of the nervous system (Damasio and Carvalho, 2013). Emotions are “action programs,” innate physiological “programs” aimed at maintaining or restoring homeostasis by changing the body to more appropriately interact with the environment (Damasio and Carvalho, 2013). Action programs are selected based on the current information the organism has on its environment and self, evaluated so as to maximize biological fitness (Rangel et al., 2008). Emotions are similar in this sense to basic instinctual drives (also action programs) such as hunger and thirst, referred to as “primordial emotions” (Denton et al., 2009). The physiological changes of action programs may be sensed by the interoceptive system and interpreted by the cortex, further influencing emotional state (Damasio and Carvalho, 2013).

Emotions are not only a key aspect of social cognition and communication, but they also initiate homeostatic physiological and cognitive functions that allow us to survive and thrive via proper detection and response to diverse challenges and opportunities (Shariff and Tracy, 2011; LeDoux, 2012). Emotional experiences are tightly bound to bodily sensations (Pace-Schott et al., 2019) and draw attention to important events such as during physiological need, immediate threat, and social interaction (Damasio and Carvalho, 2013). They coordinate behavior and physiological states during such important events (Stemmler, 2004; Nummenmaa et al., 2014). The physical effects resulting from emotional arousal are largely mediated by the autonomic nervous system (ANS), which can include changes in heart rate, skin temperature/blood flow/sweating, gut motility, pupil size, and piloerection (Kreibig, 2010; Masaoka et al., 2012; Purves et al., 2018). Bodily responses mediated by the ANS provide the most information on emotional state (Pace-Schott et al., 2019), and the ANS acts in “sympathy” with emotion, innervating the heart, lungs, and many other bodily systems (Valderas et al., 2015). Such physiological variables associated with the variety of emotions are often effortlessly measured with the right technology and have been used to detect one’s autonomic state as in the polygraph (lie detector) (Rosenfeld, 1995). While the polygraph is often inaccurate in detecting lies, it does detect arousal accurately (Lewis and Cuppari, 2009). Such changes can occur unconsciously and in response to some stimuli much quicker than changes to conscious cognition (LeDoux, 1998). Mapping the spectra of physiological, emotional triggered responses could provide an important biomarking tool for emotional state and emotional disorders.

Emotion recognition technologies may provide a basis for monitoring emotional health and could even be used to monitor for emotion-related mental health disorders (Xiefeng et al., 2019). Because the physiological changes expressed by emotions are not voluntarily controlled, they may provide a more accurate reflection of the true emotional experiences people may be having (Wu et al., 2012). The interesting relationship between emotions and the body may shed light on undiscovered processes occurring between the body and the mind. In this article, we strive to advance the use of biometrics in the personal understanding of one’s mental state by discussing the medical potential for emotion recognition devices and applications with a user-friendly emotional spectrum that one may be placed upon in a particular moment based upon visceral cues. We approach this review from a perspective of embodied cognition, following the idea that not only does emotional state significantly influence bodily state, but bodily state also significantly influences emotional state.

## Emotion, the Nervous System, and the Mind–Body Response

Emotion and bodily behaviors and sensations are linked to the point that they may be impossible to disentangle (Purves et al., 2018). Often, emotions are felt within the body (Nummenmaa et al., 2014), and these feelings occur as a result of activation of the muscular, cardiovascular, endocrine, and autonomic nervous systems (Levenson, 2006). Emotions are indeed associated with distinct bodily sensations that are culturally universal and may underlie emotional experiences, as well as corresponding to major physiological changes associated with each emotion (Ekman et al., 1983; Nummenmaa et al., 2014). We often describe our emotions in terms of bodily metaphors such as describing love as a feeling in the heart or associating fear with lowered body temperature as in the metaphor “cold feet” (Kövecses, 2000). The bodily feedback of somatic and visceral activity activated reflexively by external and internal events has been proposed to be a significant source and influencer of emotion (Barrett et al., 2007; Damasio and Carvalho, 2013; Purves et al., 2018). Voluntarily producing facial expressions or altering other bodily events such as breathing pace actually stimulates the associated emotion (Verschuuren et al., 1996) and produces the other physiological changes associated with that emotion, such as heart rate and muscle tension changes (Levenson et al., 1990).

We may understand the emotions of others by simulating them in our own minds and bodies (Niedenthal, 2007; Keysers et al., 2010; Ross and Atkinson, 2020). This simulation has been asserted to not involve conscious awareness (Wood et al., 2016). Recognizing emotional stimuli has been shown in some cases to be processed outside of conscious awareness or intent (Critchley, 2002; Philippot et al., 2002; Williams and Mattingley, 2004) and still result in the expected physiological changes (Bulut et al., 2018; Engelen et al., 2018). However, some of these results have been criticized as not generalizing to most circumstances, and there have been contradictory results. One study showed that when emotional stimuli are task irrelevant, expected muscular responses to a given emotion are not expressed (Mirabella, 2018). This may suggest that unconscious emotion recognition is not invariable and automatic but depends on the individual’s cognitive state.

The primary somatosensory cortices are engaged during the perception of emotion as well as its contagious spreading (Nummenmaa et al., 2008, 2012), and damage to them impairs the recognition of other’s emotional state (Adolphs et al., 2000). Unconscious body-state simulation of emotion-associated autonomic and visceral changes has recently been reinforced as an important aspect of understanding others’ emotions (Ross and Atkinson, 2020). Common muscular mimicry, thought to be “spillover” from simulating emotions, is abundant when observing others and occurs more to emotional stimuli (Moody et al., 2017). We suggest that respiratory pattern could be an essential mimicry target when simulating others’ emotions and that understanding how the spectrum of such bodily patterns map onto the spectrum of emotions will provide a means to measure unconscious emotion perception and emotional body-state simulation for research purposes.

Somatocentric perspectives such as the Somatic Marker Hypothesis argue that bodily states and feelings mark unconscious cognitive appraisals, which stimulate emotional experience and significantly underlie behavior and decision making. These hypotheses have been criticized, however, for being overly focused on the influence of the periphery and for misinterpretation of evidence (Dunn et al., 2006). A more moderate view we hold is that while bodily states and sensations can significantly influence the emotional mind and can act as emotional stimuli, accurate performance and decision making are not dependent on bodily markers, and the generation of emotional experience is not dependent on bodily feedback. While body states may not induce a waterfall of emotion as personal events or even music may, long-term exposure to such states may significantly alter emotional state. These body rhythms are always present with us, slowly provoking certain mental states, and so they deserve attention from not only those in health care but also the average person who would benefit from the ability to regulate emotional state. We and others have suggested that long-term “negative” body rhythms can keep us trapped in a vicious cycle of poor emotional health, which also leads to physical health declines (Jerath et al., 2019).

Positive and negative emotions differ largely in that positive emotions increase coherence of bodily rhythms, while negative emotions decrease coherence (McCraty and Zayas, 2014). A format of emotional placement based on bodily measurements may allow for the future development of more user-friendly feedback applications than currently exist that allow people to measure their emotional state and respond to it appropriately. Because the respiration–emotion relationship is reciprocal, people may voluntarily influence their emotional state by changing their respiration pattern. While emotions have such a reciprocal relationship with other bodily functions, respiration is special because it can be voluntarily altered.

## Breathing Pattern and Emotional State

Autonomic breathing is dictated not only by metabolic demands but also by emotions (Homma and Masaoka, 2008). Although it is debated whether each emotion has its own distinct signature for autonomic functions (and for other physiological mechanisms) (Kreibig, 2010), there is significant support for the idea that there is indeed some physiological specificity across the emotional spectrum (Stemmler, 2004; Nummenmaa et al., 2014), with breathing rhythm frequently labeled as an index of emotional state (Noguchi et al., 2012).

Most real-world events will induce not separate and distinct emotions but, rather, a complex mix of emotions that are most often all positive or negative (Boiten, 1998). This and the fact that full, real-world emotions are often difficult to elicit in laboratory settings make the identification of exact physiological responses and patterns of specific emotions difficult. From the available research, however, it can be concluded that more arousing responses to negative emotions (such as fear, anger, and anxiety) result in shallower, rapid breathing (Boiten, 1998; Masaoka and Homma, 2001). This may result in decreased blood carbon dioxide levels (Kreibig, 2010). Conscious modulation of breathing toward a slower and deeper pattern may strengthen positive emotions when negative emotions are prevalent (Masaoka et al., 2012). Happiness and related positive emotion produces significant respiratory changes, which include increases in the variability of the breathing pattern and decreases in tidal volume and inspiratory time (Boiten, 1998). Positive emotions vary in their effect on respiration depending on how arousing they are, with the arousing ones increasing respiration rate (Kreibig, 2010). Periods of disgust (pathogen-related) lead to suppression and cessation of breathing, likely a natural reaction of avoiding inhalation of noxious contents (Boiten, 1998).

Increasing numbers of studies show not only that emotional state influences respiratory pattern but that respiratory pattern influences and stimulates emotional state, even when one is not aware of the process (Philippot et al., 2002). The mechanism of how the body affects the mind is thought to center around modulation of the ANS and recognition of interoceptive sensation by the brain. However, respiration may act directly on the brain. Evidence is mounting showing the dramatic, seemingly direct effects respiration can have on neural oscillations across diverse brain areas (Kluger and Gross, 2020). This may synchronize neural activity and improve computational efficiency (Kluger and Gross, 2020). The respiratory rhythm has been demonstrated to unify global coordination and tuning of neural firing and dynamics across cortical and subcortical networks (Karalis and Sirota, 2018; Zaccaro et al., 2018). The respiratory rhythm can even dominate local field potentials during quiescence (Karalis and Sirota, 2018). Respiratory synchronized activity in the brain has been shown to modulate cognitive performance depending on the phase properties of the respiratory cycle (Nakamura et al., 2018). Such modulation of neural oscillations has been associated with modulations of emotion (Fumoto et al., 2004; Yu et al., 2011). Respiration can also modulate hemodynamic activity, which has a significant effect on brain activity (Başar, 2008).

## Other Responses to Emotion

While breathing is a peripheral rhythm with a special relationship with the mind, other physiological measures have emotional expression. These include cardiovascular indices, temperature, electrodermal, blood oxygenation, photoplethysmographic, and electromyographic measures (Shi et al., 2017). Although potentially invasive, biomarkers such as cortisol can also provide insight into emotional state (Strahler et al., 2017). More uncommon but revealing emotional responses including neural changes revealed by neuroimaging (Critchley and Harrison, 2013), genetic changes (Jonassen and Landrø, 2014), and inflammation (Pace-Schott et al., 2019) could be implemented into future emotion recognition technologies. Heart sounds can even be used in emotion recognition (Xiefeng et al., 2019). Heart rate variability (HRV) is the tiny variations in sinus heartbeats (Shi et al., 2017). Although some have criticized the idea, HRV is widely thought to differentiate a parasympathetic versus a sympathetic state (Balzarotti et al., 2017). HRV could thus be an important tool for identifying ANS imbalances. High HRV is sometimes associated with more positive states of mind, and HRV indices have been used in emotion recognition (Zhu et al., 2019).

Cardiac vagal control (CVC) is commonly measured by HRV, indicates the influence of the cardiovascular system on the parasympathetic nervous system (Kimhy et al., 2013), and may be an important marker for one’s ability to regulate emotion (Balzarotti et al., 2017). HRV indices may also be effective in distinguishing emotional state along with psychiatric emotional disorders (Zhu et al., 2019). Strong CVC indicates stronger vagal reactivity and recovery from stressors. Strong resting CVC is thus connected with greater ANS flexibility, cardiovascular fitness, and ability to respond to stress (Thayer and Fischer, 2009). Strong CVC is also associated with enhanced cognitive abilities such as attention, working memory, and processing speed (Hansen et al., 2003). Low HRV, and thus CVC, is associated with a variety of negative emotions (anger, sadness, and fear) and maladies including anxiety disorder, depression, cardiovascular disease, and increased risk of death (Tsuji et al., 1994; Buccelletti, 2009). Stressful events may actually lower CVC (Balzarotti et al., 2017).

Perhaps a better indicator of emotional valance is not the magnitude of the HRV but the coherence of it. Positive emotions result in a more coherent pattern, possibly providing a renewing effect. Incoherent emotions, on the other hand, produce an incoherent pattern, which is thought to have a depleting effect on health over time (McCraty and Zayas, 2014). One example of a coherence measure is the sine-like shape of heart rate patterns. Wearable devices that aim for emotion recognition should thus focus not only on basic aspects of gathered biometric data but also on patterns within and among data types.

The cardiac nervous system can act independently and is complex enough to be thought of as a “little brain” with short-term and long-term memory functions (Shaffer et al., 2014). A great portion of the fibers connecting this system to the brain are afferents, more so than any other bodily organ (Cameron, 2002). The heart is thus an intricate processing and encoding center (Armour and Ardell, 2004), which also releases its own hormones and neurotransmitters (Mukoyama et al., 1991; Huang et al., 1996). The thalamus is thought to be crucial to the formation of an integrated experience and in global cognitive functions due to its dense global networking with the cortex (Jerath et al., 2015). Research has shown that the rhythm of afferent neural information coming from the heart modulates thalamic activity, which can thus have global effects on the brain (Wölk and Velden, 1989). Frontal brain regions of the cortex (McCraty et al., 2004) as well as motor areas (Svensson and Thorén, 1979) show influence by the cardiac nervous system, and these effects include emotional processing influences (Zhang et al., 1986).

## A Spectrum of Emotion

**FIGURE 1:**
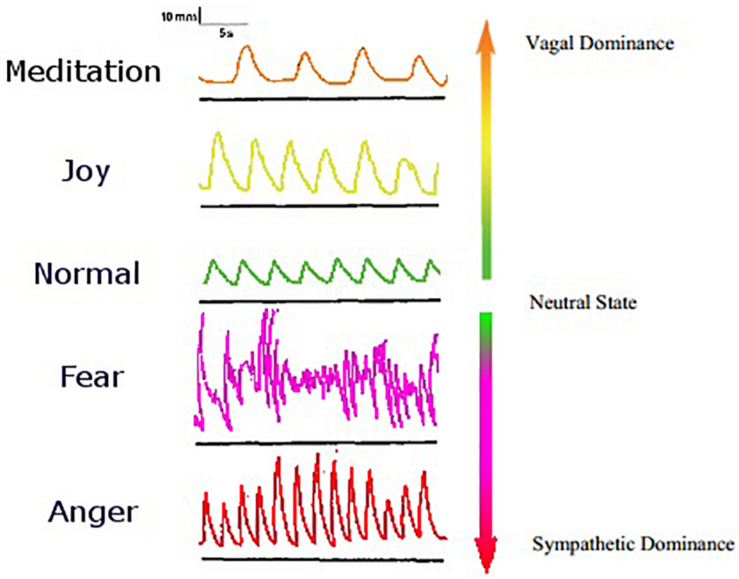
A spectrum of emotion. Affective states are shown here alongside the dimension of respiratory rhythm. The respiratory tracings shown have time on the *X*-axis and displacement on the *Y*-axis. Each respiratory state and its corresponding emotion are shown color-coded with their associated autonomic state. This spectrum is only a basic one-dimensional prototype, and future developments using the same fundamental thought could include many other biometrics for highly accurate emotion recognition. A spectrum of color is used to illustrate how biometric data may be translated into user-friendly interfaces for users of related applications and devices to quickly and easily understand their emotional and/or bodily state. A user interface that is powerful in its ability to translate biometric data into an understandable format in relation to emotion and other psychological factors will be crucial in fostering widespread and fruitful use of mind–body applications. Adapted from Kalawski (2003).

Given that emotions have largely distinct bodily patterns and are also influenced by such patterns, it may be possible to accurately map emotions along physiological dimensions. Here we give an example of this alongside dimensions of the ANS and breathing (Figure 1). Emotions are not unitary in the sense that there are different types of sadness, fear, etc., so they may be better classified for personal understanding by a color-coded spectrum. As emotions are currently regarded, single emotions sometimes have different physiological responses. For instance, sadness produces different physiological responses depending on whether it is empathetic or antipathetic (Davydov et al., 2011). Disgust is unique in that it can produce both parasympathetic and sympathetic responses depending on the nature of the reaction (pathogen vs. morality related) (Kreibig, 2010). Pathogen-related disgust can be considered more of a “primordial emotion” like thirst and hunger (Ottaviani et al., 2013). Understanding emotion via such a map will provide visual help in emotional regulation potentially by providing guidance on how to utilize the mind–body response. *Mind–body response* is a term for the psychophysiological change that occurs due to the interaction between the body and the brain, particularly focusing on the effects body rhythms can have on one’s psychology (Jerath et al., 2014). The development of such a spectrum may reveal embodiments of cognitive processes. Due to the lack of complete response specificity for basic emotions, greater dimensionality in classifying emotions may provide greater insight into their nature, reveal relationships between them, and help produce “navigational” tools for those looking to regulate their own emotions. Emotions may be regulated in a variety of means, from biofeedback methods to mind–body response techniques.

We assert that the most powerful method for emotion regulation that can be practiced by the layman pertains to aspects of the mind–body response, the most effective method being breathing techniques such as pranayama. Respiration is special in that it is not only a powerful influence on psychology and physiology but also a body rhythm that is controlled voluntarily. This puts it at the forefront of mind–body techniques.

## Conclusion

By revealing the potential nature of the intimate relationship between the body and the mind, specifically regarding emotion, we hope to pave the way for the development of new technologies and interfaces for laymen to monitor and influence their bodily state and thus mental state. Future wearable devices may utilize various physiological signs including respiration, heart rate and HRV indices, electrodermal activity, and more to recognize emotion without disrupting ongoing activities. When used in combination with machine learning techniques, personal and highly advanced recognition could be achieved. We have discussed and reviewed the nature of the emotion–body connection in order to spark innovation and insight and gave a basic example of a physiological spectrum of emotion for use in wearable devices to provide user-friendly and easily understandable interfaces for consumers to assist them in identifying, regulating, and modifying their emotional state.

## Author Contributions

RJ developed the theory. CB wrote the manuscript. Both authors did the literature review and contributed to the article and approved the submitted version.

## Conflict of Interest

The authors declare that the research was conducted in the absence of any commercial or financial relationships that could be construed as a potential conflict of interest.
